# Lactulose as a prebiotic improved the intestinal health and metabolism of geese

**DOI:** 10.3389/fvets.2025.1721442

**Published:** 2025-12-09

**Authors:** Xiaoxue Wang, Pengfei Ye, Jie Zhu, Xueqi Zhu, Xiaorong He, Yu Meng, Shiyan Cao, Lei Zhao

**Affiliations:** 1 College of Animal Science, Anhui Science and Technology University, Chuzhou, China; 2 Anhui Province Key Laboratory of Animal Nutritional Regulation and Health, Anhui Science and Technology University, Chuzhou, China

**Keywords:** lactulose, growth performance, uric acid, intestinal morphology, intestinal flora, metabolites

## Abstract

Lactulose is highly valued for its unique role in promoting the growth of intestinal probiotics. The objective of this research was to investigate how varying levels of dietary protein combined with lactulose supplementation influence the intestinal health of geese, utilizing intestinal metabolomics as the analytical basis. A total of 210 one-day-old Yangzhou geese were randomly assigned to three dietary treatments, with each group consisting of 7 replicates of 10 birds each. The diet of the control group (CP) contained 18.18% crude protein, the high-protein group (HP) was provided a diet formulated to 21.12% crude protein, and the lactulose-supplemented group (LS) received the high-protein diet (21.12%) with an additional 0.30% lactulose inclusion. The results showed that compared with the HP group, lactulose increased the average daily weight gain of geese (*p* < 0.05), reduced the feed conversion rate (*p* > 0.05), and decreased the level of uric acid in serum (*p* < 0.05). At the same time, lactulose improved the morphological structure of the ileum and increased the intestinal villus height (VH) (*p* < 0.01) and villus height/crypt depth ratio (VH/CD) (*p* < 0.05). Compared with HP, the total number of aerobic bacteria, *Escherichia coli* and *Salmonella* in cecum decreased by 2.70, 9.28 and 12.26%, respectively. Non-targeted metabolomics analysis showed that lactulose regulated intestinal barrier structure and intestinal flora through glycerophospholipid metabolism and fatty acid metabolism, which further improved the intestinal health of geese.

## 1 Introduction

In poultry production, the proper functioning of the intestinal tract is critically linked to overall health and productivity. As the largest immune organ and a vital barrier against pathogens, the intestine plays a fundamental role in maintaining systemic homeostasis (1). Its integrity and physiological balance directly influence the immune defense capacity, disease resistance, and overall production performance of poultry. Biological systems generally have a defined two-way network system, that is, the intestinal axis (covering intestinal-brain, microbiota-immune, nerve-immune and other connections) (2). In animals, intestinal diseases and many other pathological conditions are often caused by ecological disorders. With the promotion of large-scale breeding mode, the density of goose farming has increased, and geese are vulnerable to stress, diet and other factors, resulting in decreased resistance, intestinal flora imbalance and gastrointestinal diseases. These challenges not only compromise goose health but also hinder the sustainability of the goose farming industry. With the prohibition of antibiotics, alternative feed additives have been increasingly adopted to enhance goose health. Among these, prebiotic products such as oligosaccharides have gained prominence and are now extensively utilized in poultry nutrition as high-quality prebiotics (3). Lactulose is a high-quality oligosaccharide with a variety of biological functions. Lactulose resists intestinal digestion and absorption, functioning as a fermentable substrate for commensal gut microbiota to generate bioactive metabolites (4). These metabolites can provide energy for intestinal cells, promote intestinal peristalsis, and maintain intestinal patency. Furthermore, lactulose promotes intestinal homeostasis through modulation of gut microbiota composition, suppression of pathogenic bacterial proliferation (5), and enhancement of mucosal immune function (6).

Metabolomics can comprehensively analyze metabolites in organisms and reveal the metabolic characteristics of organisms under different physiological or pathological conditions (7). Metabolomics technology has been widely used in animal nutrition and health field (8). Wang et al. (9) employed non-targeted metabolomics to analyze rumen metabolites in dairy cows, demonstrating that inulin supplementation significantly modulates amino acid and lipid metabolic pathways. The non-targeted metabolomics analysis of the ileum and duodenum of green shell ducks infected with duck enteritis virus (DEV) by Cai et al. (10) showed that tryptophan level and fat metabolism were related to inflammation and immune response.

Although the role of lactulose in animal intestinal health has been preliminarily verified, its specific metabolic process in geese and its detailed mechanism of influence on intestinal health are still unclear. Therefore, this study intends to use intestinal metabolomics technology to reveal the metabolic characteristics of goose lactulose and explore its potential impact on intestinal health by comparing and analyzing the changes of metabolites in high protein group, blank control group and lactulose intake group. This study not only contributes to a deeper understanding of lactulose’s effects on the metabolic processes in geese, but also provides new perspectives and approaches for regulating intestinal health in geese.

## 2 Materials and methods

### 2.1 Materials and methods

The animal experimental design was approved by the Animal Care and Use Committee of Anhui Science and Technology University. The test animals were provided by Huaxin Poultry Company (Anhui, China). Two hundred and ten healthy one-day-old Yangzhou goslings with similar initial body weights were selected for the experiment. The feeding period was 42 days (from 2023-10-18 to 2023-11-29). The geese were randomly allocated to the experimental treatments following a completely randomized design. The trial consisted of several dietary treatment groups, each comprising seven replicate pens containing 10 birds. The crude protein content in CP feed was 18.18%, HP was fed with isonitrogenous formula feed (crude protein content 21.12%). Previous studies have shown that adding low doses of lactulose to poultry is most conducive to the growth and development of poultry (11). LS and HP had the same protein content and added 0.30% lactulose. Goose was allowed to drink water freely throughout the trial period. The basal diet ratio was adjusted according to the latest research of Abou-Kassem et al. (12) to better meet the needs of modern commercial geese under intensive feeding conditions. The specific ingredient composition and nutritional content of the diets are detailed in Table 1.

**Table 1:** Composition and nutritional level of basic diet (air-dry basis).

Items	Content (CP)	Content (HP)
Ingredients		
Corn	56.00	50.00
Soybean meal	5.00	15.00
Rice bran	4.40	4.50
Fish meal	2.00	1.00
Wheat bran	17.50	14.60
Cottonseed meal	5.00	4.00
Limestone	0.60	0.70
CaHPO_4_	0.10	0.10
DL-methionine	0.20	0.20
Corn protein meal	7.20	7.90
NaCl	1.00	1.00
Premix^a^	1.00	1.00
Total	100	100
Nutrient levels^b^		
ME, MJ/kg	11.25	11.21
Crude protein, %	18.18	21.12
Crude fat, %	4.29	4.09
Crude fiber, %	4.00	4.12
Ca, %	0.40	0.42
Total phosphorus, %	0.63	0.62
Lys	0.65	0.84
Met	0.56	0.59

a Per kg of premix contains: vitamin D 1,000 IU, vitamin A 4,500 IU, vitamin E 30 IU, vitamin K3 1.3 mg, vitamin B 12.2 mg, vitamin B2 10 mg, vitamin B12 1.013 mg, vitamin B6 4mg, Ca 7.5 mg, niacin 20 mg folic acid 0.5 mg, bio-tin 0.04 mg, Cu 7.5 mg, Fe 60 mg, Zn 65 mg, Mn 110 mg, I 1.1 mg, Se 0.15 mg.

b Crude protein is measured values, and other nutrients are calculated values.

### 2.2 Growth performance

The geese were weighed on days 1, 7 and 42 of the experiment. Feed intake (FI) was recorded daily during the experiment. At 42 days of age, the average daily gain (ADG) and feed conversion rate (FCR) of each group were measured.

### 2.3 Biochemical profile

At 42 days of age, the blood was collected from the jugular vein of the goose after 12 h of fasting (6 in each group). About 1–2 mL of blood was drawn from each goose, and the serum was centrifuged for subsequent analysis. Quantification of serum uric acid, creatinine, and blood urea nitrogen concentrations was performed employing standardized assay kits provided by Nanjing Jiancheng Bioengineering Institute. Serum biochemical indicators were operated according to the kit steps. Uric acid content was determined by enzyme colorimetry. The creatinine content was determined by microplate method. The urea nitrogen content was determined by urease method.

### 2.4 Morphology analysis of ileum

Geese were humanely euthanized following CO_2_-induced anesthesia and cervical dislocation, in compliance with institutional animal ethics guidelines. Duodenum, jejunum, ileum, cecum and contents were separated. Mid-ileal segments (2 cm) were excised, fixed in 4% paraformaldehyde (PFA), and processed for morphology analysis following standard protocols (6 in each group). Morphology analysis was embedded by paraffin embedding technique. The standard paraffin embedding process was used in the experiment. The thickness of the tissue section after embedding was 5 μm. The tissue sections treated by hematoxylin–eosin staining were finally sealed with neutral resin. ImageJ software was used for observation and image interception. Villus height and crypt depth measurements were performed on at least five morphologically preserved villi per slide, ensuring consistency across all samples.

### 2.5 Intestinal microbial populations

Cecal contents were used for microbial flora analysis (6 in each group). The cecal contents were removed from the refrigerator, and the cecal microorganisms were determined by plate coating method after natural thawing. The experimental procedure is as follows: the sample (0.1 g) was homogenized with 0.9 mL of sterile PBS buffer to obtain a 10^−1^ dilution. After homogenization, 0.1 mL supernatant was taken and diluted to 10^−2^ suspension in 0.9 mL sterile PBS buffer test tube. Gradually diluted to 10^−3^, 10^−4^, 10^−5^, 10^−6^, 10^−7^ for plate culture. Bacterial cultivation protocols included: (1) anaerobic growth of *Lactobacillus* in MRS agar (37 °C, 48 h), (2) Total aerobic bacterial enumeration on TTC nutrient agar, (3) *Salmonella* enumeration on HE agar, and (4) *Escherichia coli* detection via MacConkey agar, with the latter three incubated aerobically at 37 °C for 24 h. For analytical consistency, total bacterial counts in intestinal contents were converted to common logarithmic values and expressed as log₁₀(CFU/g).

### 2.6 Metabolomics analysis

The ileum tissue was rinsed with normal saline and frozen for metabolomics analysis (6 in each group). Frozen samples (−80 °C) were thawed under controlled ambient conditions, homogenized using sterile blades, and subsequently aliquoted into pre-labeled centrifuge tubes following precise gravimetric measurement (20 ± 1 mg). The microstructure and steel balls were homogenized for 20 s and centrifuged 30 s. Following centrifugation, 400 μL of a 70% methanol–water solution containing internal standard was added to each sample, followed by vortex mixing for 5 min. After equilibration in an ice bath for 15 min, the samples were subjected to an initial centrifugation step (12,000 × g, 10 min, 4 °C). A 300 μL aliquot of the resulting supernatant was then transferred into pre-chilled, uniquely coded vials and frozen at −20 °C for 30 min. A second centrifugation was performed under the same conditions to complete the preparation. Finally, the supernatant was collected for subsequent online analysis by LC-MS.

### 2.7 Data statistics and analysis

All analyses in this study were performed using SPSS 20.0 software. One-way ANOVA analysis of variance combined with *post-hoc* test was used to clarify the significant differences. All the data were checked for normality. Duncan multiple comparison method was used to compare the mean values, and the significance level was set as *p* < 0.05. Data are presented as the mean ± standard error (SE). Origin 2018 software was used to make histograms for comparative analysis. Based on the bioinformatics platform of KEGG database, pathway enrichment analysis was conducted on the identified differential metabolites to functionally annotate and interpret their involved metabolic pathways.

## 3 Results

### 3.1 Growth performance

As shown in Table 2, geese subjected to long-term high-protein diet exhibited reductions in final body weight (FBW), ADG, and FI compared to those in the CP group. Compared with HP, lactulose increased the FBW and ADG of geese (*p* < 0.05).

**Table 2:** Effects of different diets on growth performance of geese.

Item	CP	HP	LS
IBW (g)	379.44 ± 7.69	350.07 ± 8.06	362.72 ± 7.77
FBW (g)	2489.25 ± 19.37^bc^	2456.04 ± 67.17^c^	2701.95 ± 49.69^a^
ADG (g)	60.28 ± 0.34^b^	60.17 ± 1.89^b^	66.84 ± 1.51^a^
FI (g)	4956.8 ± 51.87^b^	5165.33 ± 94.01^b^	5539.76 ± 102.74^a^
FCR	2.35 ± 0.01	2.47 ± 0.10	2.38 ± 0.08

IBW, initial body weight; FBW, final body weight; ADG, average daily gain; FI, feed intake; FCR, feed conversion ratio. ^a,b,c^Means in the same row with unlike superscripts were significantly different (*p* < 0.05).

### 3.2 Biochemical profile

**Figure 1:**
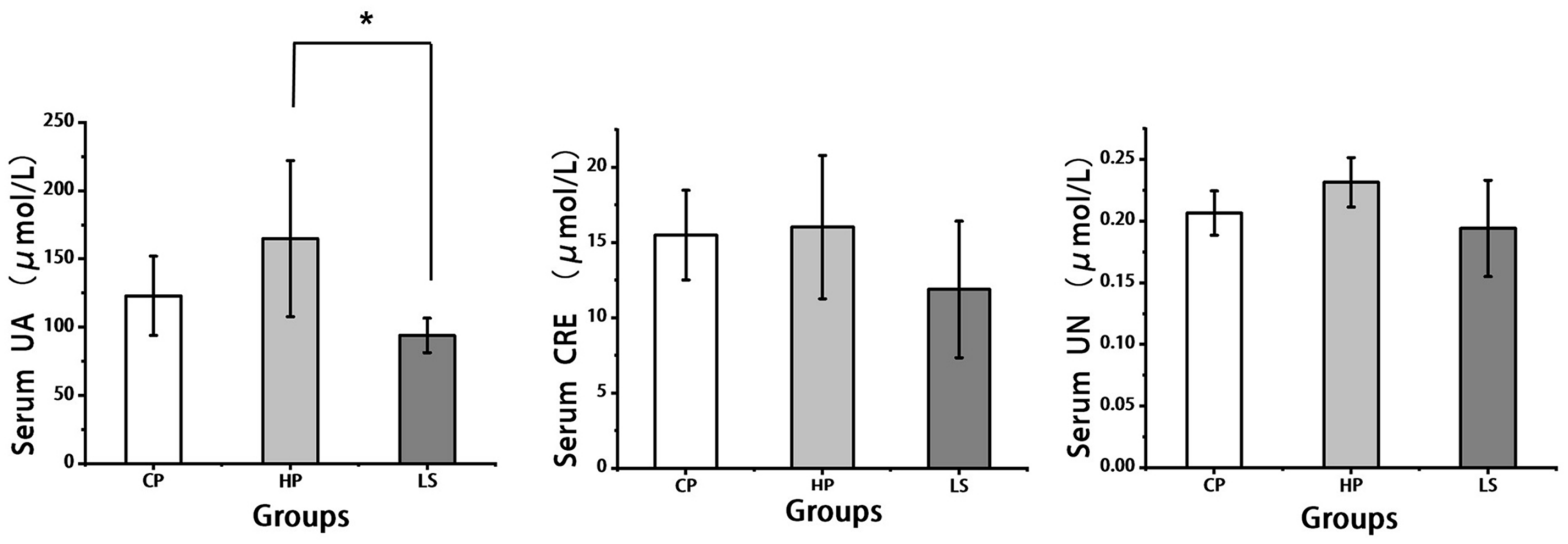
Effects of different diets on serum biochemical indexes of geese.

As illustrated in Figure 1, dietary supplementation with lactulose significantly reduced serum uric acid levels (*p* < 0.05) in geese compared with the high-protein group, while no statistically significant differences were observed in creatinine or urea nitrogen levels between the two groups. However, numerically, lactulose reduced the content of these two indicators compared to HP.

### 3.3 Morphology analysis of ileum

**Figure 2:**
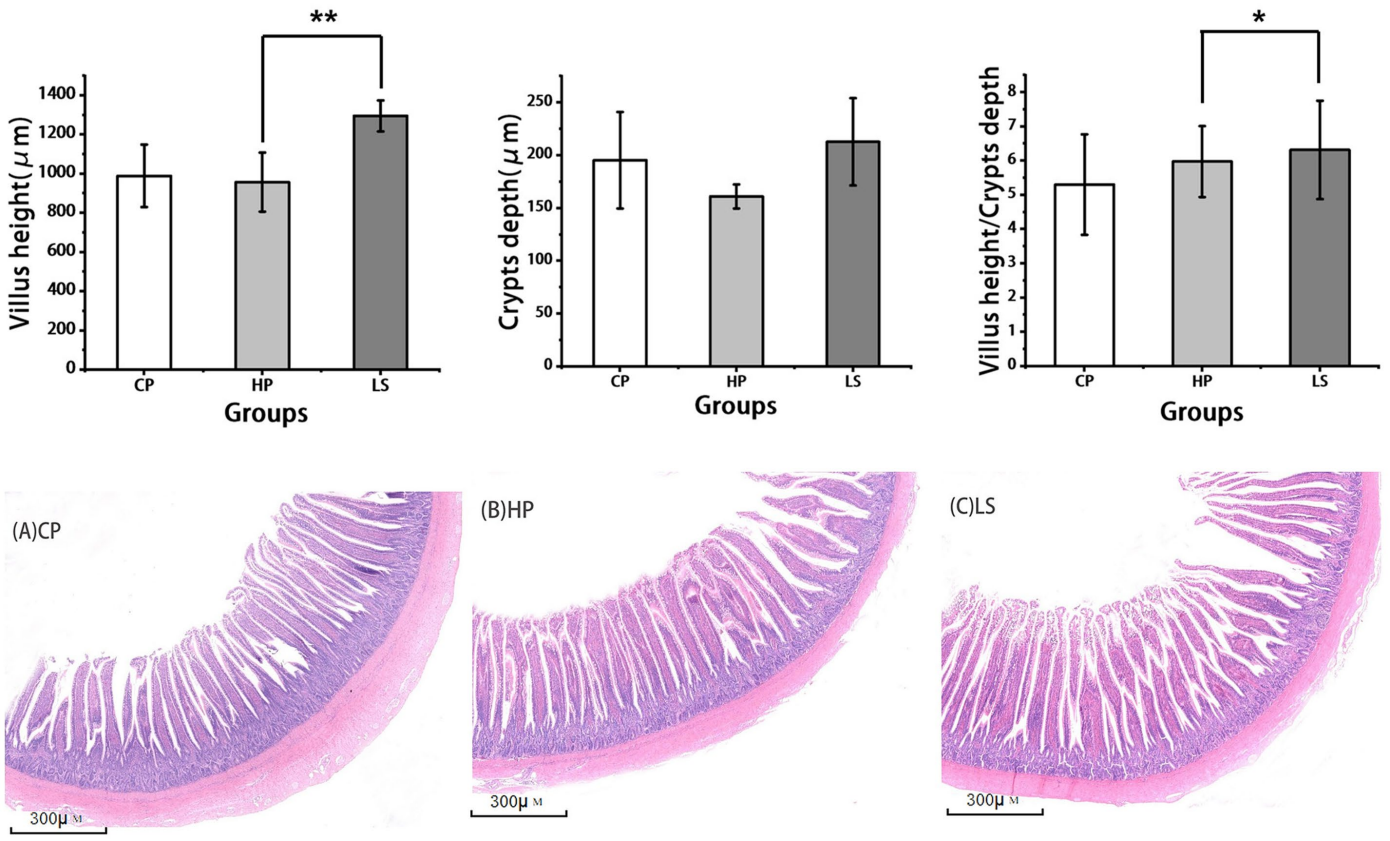
Effects of different diets on intestinal morphology of geese. (**A–C** is the slice diagram of goose ileum, 40×).

As shown in the histological sections in Figure 2, a high-protein diet resulted in shortened intestinal villi in geese. In contrast, the inclusion of lactulose in the diet effectively restored villus height to a level comparable to that of the normal control group. Compared with HP, lactulose increased ileal VH (*p* < 0.01) and VH/CD (*p* < 0.05).

### 3.4 Intestinal microbial populations

As shown in Table 3, compared to the basal diet, the high-protein diet significantly increased the intestinal abundance of *Escherichia coli* (*E. coli*) (*p* < 0.01) and *Salmonella* (*p* < 0.05), while it decreased the abundance of *Lactobacillus* (*p* < 0.05). In contrast, adding lactulose to a high-protein diet reduced the growth of *E. coli* and *Salmonella* (*p* < 0.01).

**Table 3:** Effects of different diets on cecal microflora of geese.

Item	CP	HP	LS
Total aerobic bacteria	8.47 ± 0.01^a^	7.42 ± 0.01^b^	7.22 ± 0.06^c^
*Escherichia coli*	5.92 ± 0.01^Bc^	6.79 ± 0.04^Aa^	6.16 ± 0.04^Bb^
*Salmonella*	5.09 ± 0.02^c^	6.77 ± 0.01^Aa^	5.94 ± 0.01^Bb^
*Lactobacillus*	8.09 ± 0.06^a^	7.40 ± 0.01^b^	7.38 ± 0.03^b^

CP, control group (basal diet); HP, high-protein group (high-protein diet); LS, lactulose group (HP + 0.3% lactulose). Data represent mean values of 6 replicates per treatment. ^a,b,c^Means in the same row with unlike superscripts were significantly different (*p* < 0.05). ^A,B,C^Means in the same row with unlike superscripts were significantly different (*p* < 0.01).

### 3.5 Intestinal metabolomics analysis

**Figure 3:**
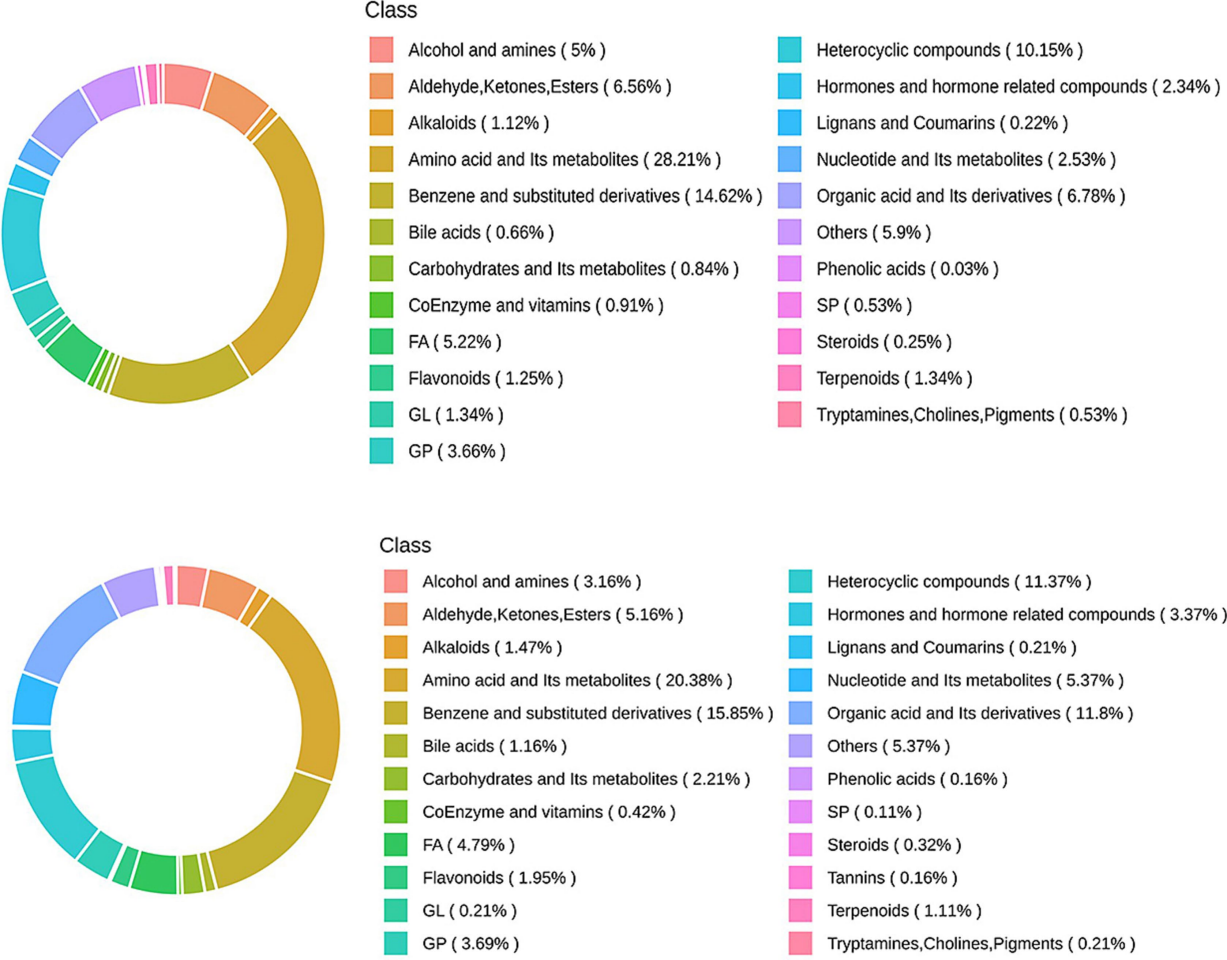
Metabolites of positive and negative ions.

Figure 3 illustrates the total metabolites detected under both positive and negative ionization modes. In this study, we analyzed 18 goose ileum samples, which were categorized into three distinct groups for a comprehensive metabolic assessment, yielding a total of 4,674 metabolites. All the metabolites were classified and analyzed. Metabolite profiling identified several major classes of compounds under each ionization mode. In positive ion mode, the predominant metabolites included amino acids and their metabolites (28.21%), benzene and substituted derivatives (14.62%), heterocyclic compounds (10.51%), organic acids and derivatives (6.78%), esters (6.56%), as well as nucleotides and related metabolites (2.53%). In negative ion mode, the composition was primarily characterized by amino acids and their metabolites (20.38%), benzene and substituted derivatives (15.85%), organic acids and derivatives (11.80%), and heterocyclic compounds (11.37%), among others.

**Figure 4:**
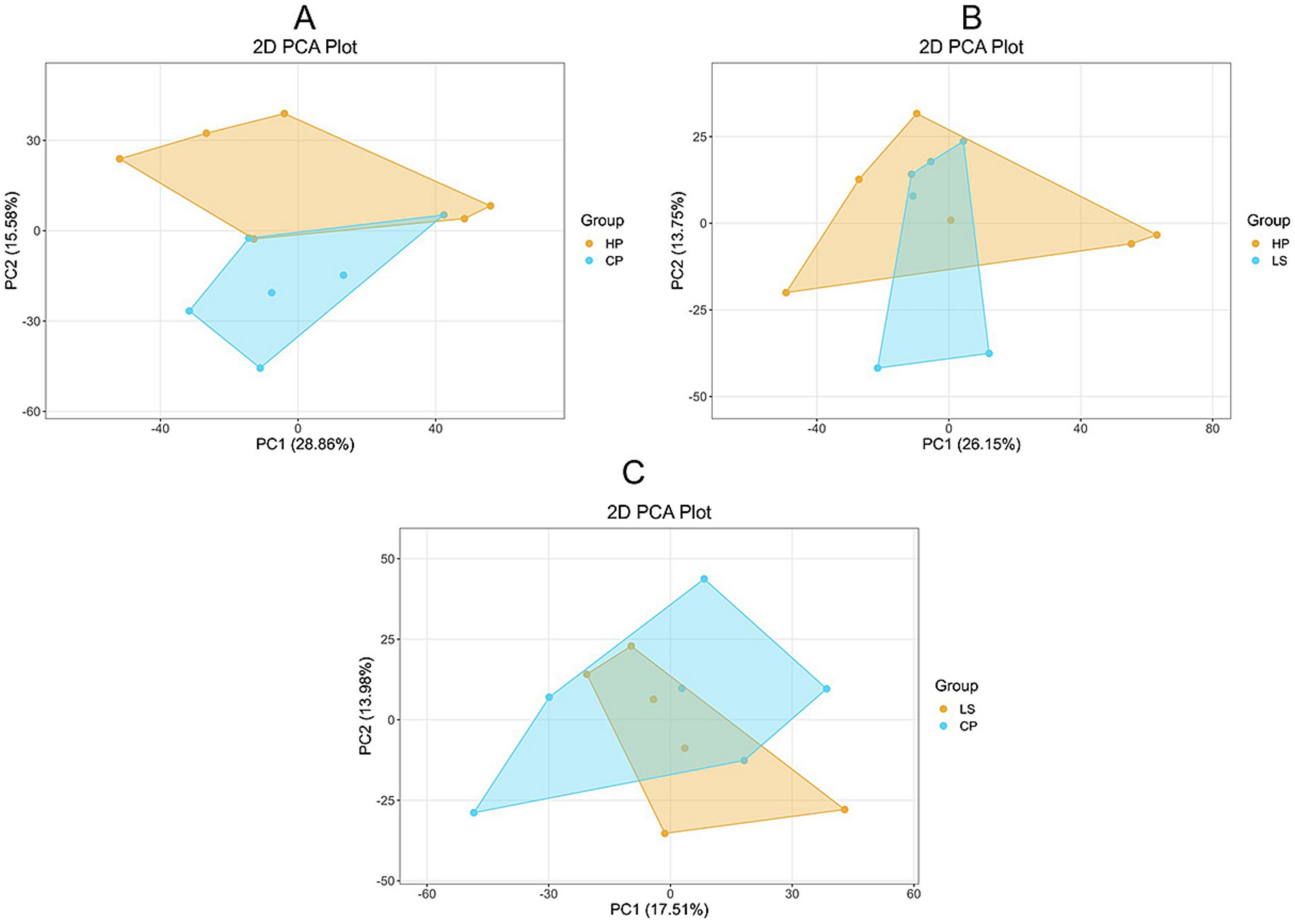
Pairwise comparison of PCA analysis results.

Pairwise comparison PCA analysis was performed on CP, HP and LS samples using non-targeted LC–MS metabolomics analysis (Figure 4). In the PC1 and PC2 dimensional maps, there is a certain separation trend between the groups of samples. The data is reliable and can be used for further analysis.

**Figure 5:**
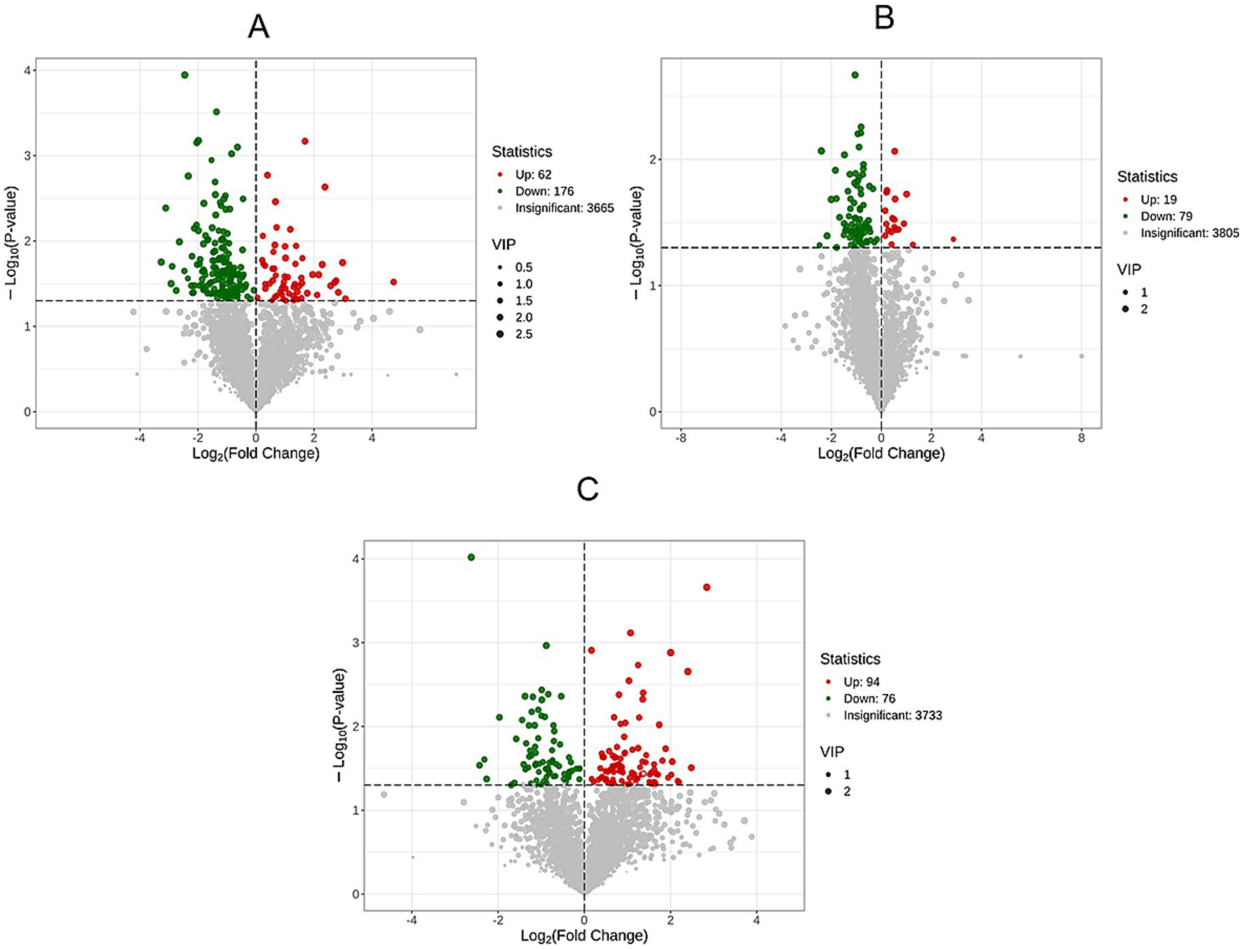
Volcanic maps of differential metabolites between groups.

Volcanic maps of differential metabolites were drawn according to the following criteria: VIP >1.0, *p* < 0.05 (Figure 5). Data analysis showed that there were 238 differentially expressed metabolites in HP and CP, of which 62 were significantly up-regulated and 176 were significantly down-regulated (Figure 5A). Between HP and LS, 19 differential metabolites were significantly up-regulated and 79 differential metabolites were significantly down-regulated (Figure 5B). There were 94 differential metabolites significantly up-regulated and 76 differential metabolites significantly down-regulated between LS and CP (Figure 5C).

**Figure 6:**
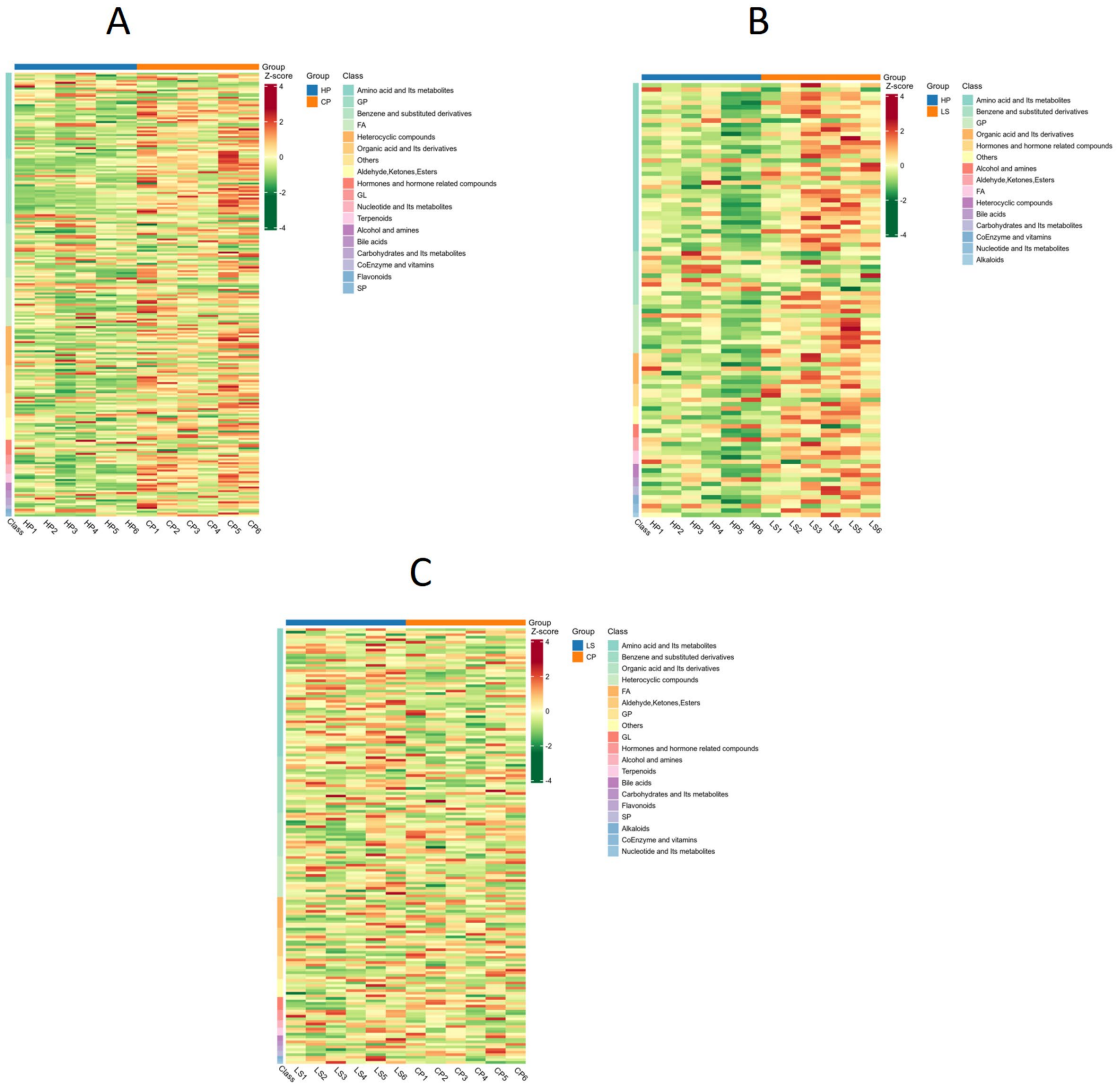
Metabolic heat map between groups.

Through the heat map, the expression pattern of metabolites between groups can be visualized (Figure 6). Amino acids and their metabolites, benzene and substituted derivatives, as well as organic acids and their derivatives were identified across the HP, CP, and LS groups. Compared with the HP group, the LS group exhibited increased abundances of amino acids and their metabolites, along with organic acids and their derivatives.

**Figure 7:**
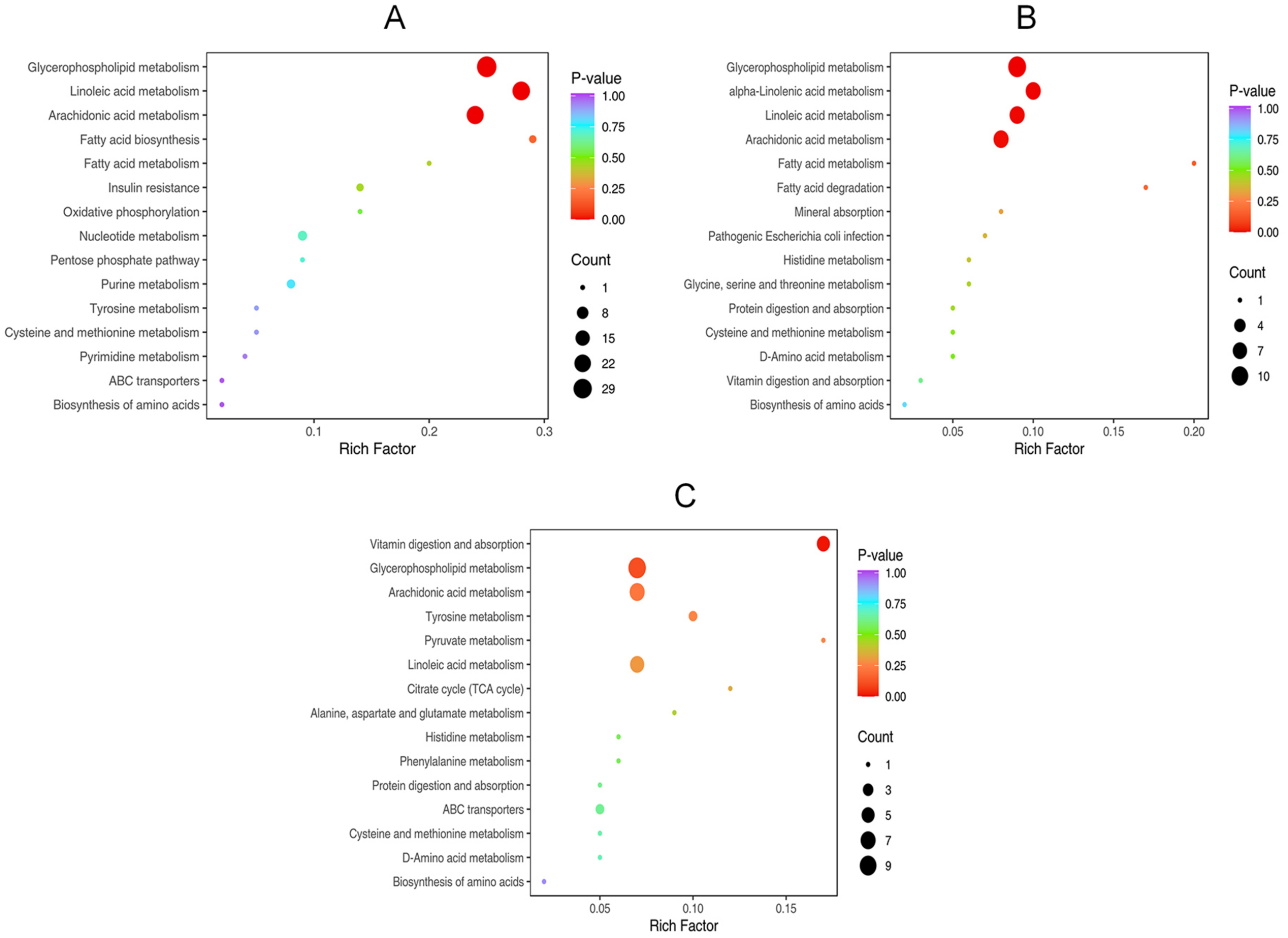
KEGG enrichment analysis of differential metabolites.

As summarized in Figure 7, pathway enrichment analysis based on the KEGG database revealed the significantly altered metabolic pathways associated with the differential metabolites. In the HP VS. CP, the enriched pathway items were mainly Purine metabolism, fatty acid-related and metabolic pathways. In the HP VS. LS, the enriched pathways were primarily related to glycerophospholipid metabolism, fatty acid metabolism, and amino acid metabolism. In the LS VS. CP, differential metabolites were mainly annotated and enriched in organic acid metabolism, amino acid metabolism, such as alanine, aspartic acid and glutamic acid metabolism, cysteine and methionine metabolism, and histidine metabolism.

**Figure 8:**
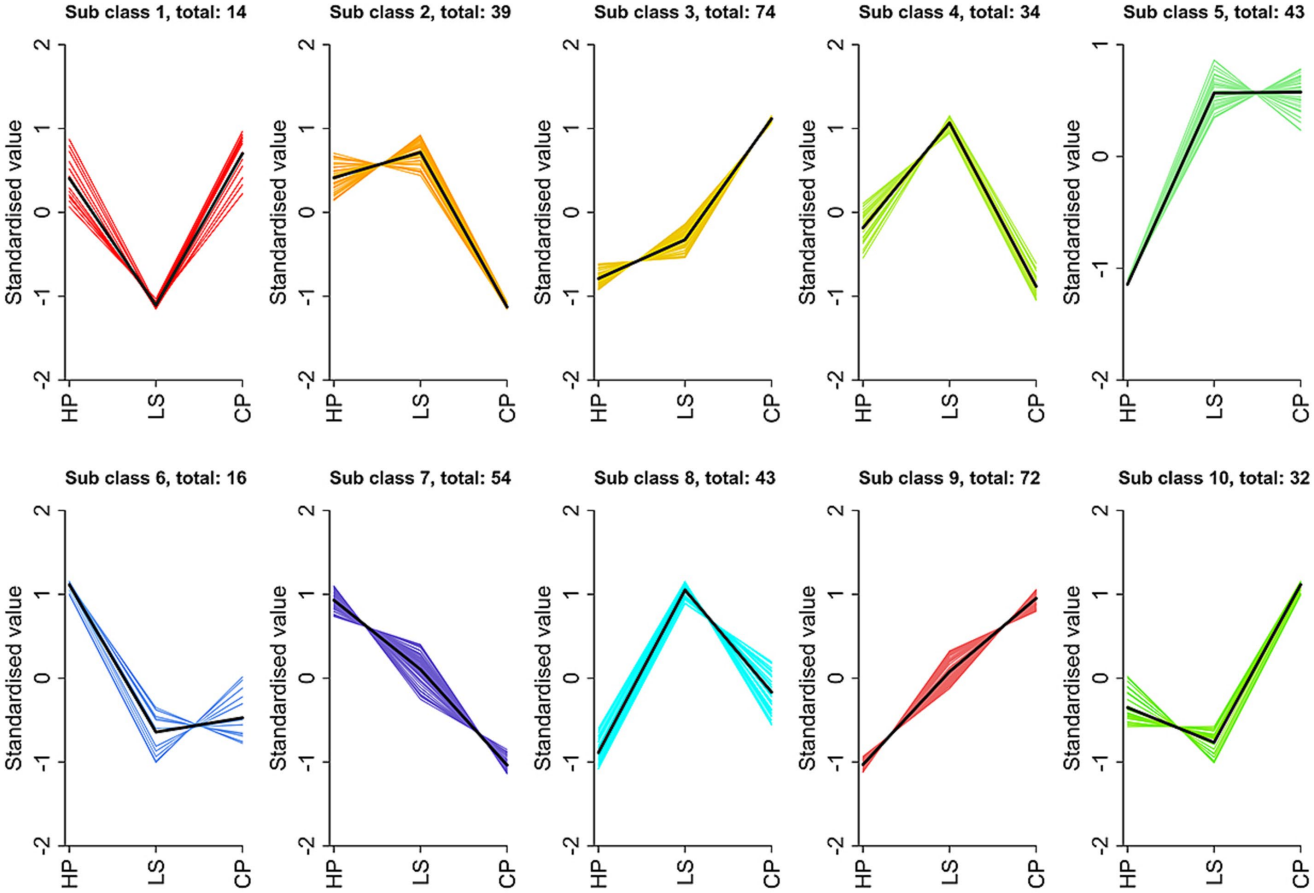
k-means clustering analysis.

Figure 8 displays the variation trends in relative metabolite abundance across the experimental groups as determined by K-means cluster analysis. We have obtained a total of 10 trend sets. Among them, subclass 3 and subclass 8 are the two subclasses we pay more attention to, and they are more in line with our expected results. In sub-class 3, metabolites decreased from CP to HP, and gradually increased in LS. The components are mainly carnitine, glutamic acid, arginine and tyrosine. In sub-class 8, the metabolites showed an upward trend from HP to LS. Carnitine, glycine and lysine are the main components.

## 4 Discussion

### 4.1 Growth performance

The production performance of geese is not only the core index to measure the breeding efficiency, but also the key fulcrum to affect the sustainable development of the whole industrial chain. In this study, lactulose increased the ADG and FI of geese. Previous research has consistently indicated that dietary supplementation with lactulose yields beneficial outcomes in terms of growth performance in both poultry and swine. A growing body of evidence further corroborates its efficacy in broiler production. For instance, Calik and Ergün (4) reported a statistically significant increase in final body weight following lactulose administration. Similarly, Zhao et al. (13) observed not only elevated body weight gain but also an improved feed conversion ratio in broilers receiving lactulose. These collective findings imply that lactulose may enhance growth metrics by positively influencing intestinal health, thereby promoting more efficient nutrient absorption and utilization. Similarly, research conducted on weaned piglets has demonstrated the beneficial effects of dietary lactulose supplementation. Guerra-Ordaz et al. (14) demonstrated that the dietary inclusion of lactulose led to significant improvements in several key growth performance metrics, including enhanced feed intake, greater average daily gain, and a more efficient feed conversion ratio. These findings align with the outcomes of our study. The palatability of lactulose increased the feed intake of geese. Although the precise mechanism through which lactulose enhances growth performance in geese remains incompletely elucidated, it is hypothesized to be associated with positive modifications in the composition of the intestinal microbiota and the reinforcement of the intestinal barrier function, as suggested by Calik and Ergün (4). In summary, adding 0.50% lactulose to the feed is a cost-effective strategy to optimize the growth performance of meat geese.

### 4.2 Biochemical profile

The concentration changes of uric acid, urea nitrogen and creatinine in goose serum can directly reflect the nitrogen metabolism efficiency and the functional status of the excretory system. As an important place for nitrogen metabolism, the health status of the intestine has a close interaction with these indicators. In this study, lactulose reduced serum uric acid levels. Bai et al. (15) also obtained similar results. The underlying mechanism for lactulose’s serum uric acid-reducing effect may involve its resistance to small intestinal digestion. This allows it to transit to the colon, serving as a substrate for metabolism by the resident intestinal flora. Lactulose has been shown to decrease uric acid levels through inhibition of urease-producing bacterial growth in the intestine, thereby reducing ammonia production (16). Furthermore, lactulose supplementation was found to lower serum urea nitrogen concentrations. When the intestinal barrier is damaged, endotoxin (LPS) enters the blood to activate the systemic inflammatory response, which will aggravate the accumulation of urea nitrogen (17). Lactulose can indirectly reduce the level of urea nitrogen in goose serum by repairing intestinal mucosal barrier (18). These results suggest that lactulose may affect the nitrogen metabolism of geese by improving intestinal health.

### 4.3 Morphology analysis of ileum

The structure of intestinal mucosa can directly reveal the degree of intestinal health. In morphological studies, the assessment of intestinal health relies heavily on two key parameters: villus height and crypt depth. Villus height is directly correlated with the absorptive capacity and barrier function of the gut, as taller villi provide a greater surface area and a more robust physical barrier against intraluminal pathogens and toxins (19). Conversely, crypt depth is indicative of the rate of epithelial cell renewal and repair. Extended intestinal villus height enhances mucosal absorptive capacity, whereas reduced crypt depth reflects diminished epithelial turnover and attenuated demands for cellular proliferation (20). Consistent with our findings, dietary lactulose supplementation in this study significantly improved intestinal morphology in geese, as evidenced by increased villus height (VH) and an elevated VH/CD ratio in the ileum. Similar effects have been reported in other poultry species; for instance, Chang et al. (21) observed that the inclusion of oligosaccharides at 30 mg/kg in the diet of yellow-feathered broilers also enhanced intestinal villus height and the VH/CD ratio. According to the findings of Xu et al. (22), the administration of fructooligosaccharides in the diet at a concentration of 4.0 g/kg was demonstrated to significantly enhance ileal villus height in broilers. Mohammadi Gheisar et al. (11) have shown that adding low-dose lactulose to the diet of laying hens can have a beneficial effect on the intestine, which is manifested by an increase in intestinal villus length, an increase in intestinal crypts, and an increase in muscle thickness. This morphological change suggests a potential improvement in the gut’s absorptive capacity and barrier function. This positive effect was likely achieved through modulation of the gut microbiota. It can be hypothesized that the improvement in intestinal morphology may be attributed to lactulose-mediated beneficial modulation of gut microbiota, thereby regulating the differentiation and proliferation of intestinal epithelial cells.

### 4.4 Intestinal microbial populations

Intestinal microflora is a resident microorganism in the digestive tract of animals, which affects the digestion of nutrients and the biotransformation of food compounds in host organisms. In this study, microbial culture-based methods were employed to quantify total aerobic bacteria, *E. coli*, *Salmonella*, and *Lactobacillus* in the cecal contents. The results indicated that a high-protein diet promoted the proliferation of harmful bacteria, such as *E. coli* and *Salmonella*, in the intestinal tract of geese. In contrast, dietary supplementation with lactulose effectively suppressed the growth of these pathogenic bacteria. Xi et al. (23) also obtained similar results. Long-term feeding of high-protein diet during the gosling period will lead to intestinal microecological imbalance and increase the proliferation of harmful bacteria. Lactulose is a high-quality prebiotic. Adding prebiotics to feed can improve intestinal flora and produce metabolites that may protect intestinal function (24). The ability of lactulose to suppress pathogen proliferation is mediated through its pH-lowering effect. By undergoing fermentation, it releases short-chain fatty acids that acidify the intestinal mucosa (25). This resulting acidic environment selectively inhibits harmful bacteria and supports overall intestinal development. The growth of common pH-sensitive pathogens such as *Escherichia coli* and *Salmonella* becomes significantly inhibited when environmental acidity reaches pH levels under 5, while acid-resistant microbial strains demonstrate sustained viability under these conditions (26). The decline in harmful intestinal bacteria may be attributed to pH modulation, echoing similar findings established in prior literature (13). However, the potential concern was that the number of lactobacilli did not increase in the treatment group fed the lactulose diet. This observed outcome may be attributable to the specific concentration of lactulose administered, highlighting the need for further investigation that specifically examines the dose-dependent effects of lactulose on the composition and metabolic activity of the intestinal microbiota.

### 4.5 Intestinal metabolomics analysis

As a core component of the body’s digestive system, the intestine plays a vital role, and its functions are diverse and complex. Intestinal metabolism is fundamental to gut homeostasis, pivotal in preserving barrier integrity and modulating mucosal immunity. To further elucidate the influence of intestinal metabolites on gut health and systemic physiological development, we employed metabolomic profiling to analyze ileal tissue. The analytical results demonstrated a strong correlation between intestinal health status and alterations in multiple metabolite categories, notably amino acids and their derivatives, organic acids and associated compounds, and glycerophospholipids. Deng et al. (27) obtained similar results, and they detected these substances in cecal contents of geese.

Next, we analyzed the differential metabolites between different groups. Interestingly, compared with the normal diet, the down-regulated metabolites in the high-protein diet group were more than the up-regulated metabolites. Most types of carnitine are down-regulated metabolites, such as carnitine C16-OH, carnitine C8-OH, carnitine C19. Enterobacteriaceae use γ-butyrobetaine as a substrate to produce carnitine. The carnitine transporter Octn2 is expressed specifically in the epithelial cells lining both the small and large intestine, where it mediates the uptake of dietary carnitine (28). According to Shekhawat et al. (29), deficiency in carnitine results in significant intestinal impairment, including mucosal atrophy, ulceration, and inflammatory damage in animal models. Elevated levels of acetyl-L-carnitine promote fatty acid *β*-oxidation, a metabolic process crucial for supporting the production of B cells and T cells, thereby facilitating immune defense against infection and injury (30). Diet regimens with increased protein intake showed a significant decrease in carnitine concentration, indicating a potential adverse effect on intestinal homeostasis. And combined with the data of intestinal morphology, the intestinal villi of the high protein group became shorter, which also confirmed this. Lactulose was added to the high-protein diet, and the content of carnitine in animals increased. It can be seen that lactulose can promote the production of B cells and T cells to resist infection.

The KEGG pathway enrichment analysis revealed that glycerophospholipid metabolism and fatty acid metabolism were the primary pathways enriched by metabolites following the supplementation of a high-protein diet with lactulose. As key structural components of both the tight junctions between intestinal epithelial cells and the mucus layer, glycerophospholipids contribute to the preservation of intestinal barrier integrity, support a healthier gut microbial community, and play a role in the regulation of intestinal immunity (31). Glycerophospholipids have emerged as promising candidate diagnostic biomarkers for specific intestinal pathological states. Their characteristically altered metabolic profiles in diseases such as inflammatory bowel disease (IBD) offer significant potential for non-invasive diagnostic applications. Phosphatidylcholine (PC) content increased in glycerophospholipid metabolism. Phosphatidylcholine is a vital constituent of the gut’s protective mucus layer. It supports barrier function both structurally, by binding to mucins to generate mucus, and biologically, by exerting direct anti-inflammatory effects on the mucosal tissue. This dual role is instrumental in alleviating conditions like inflammatory bowel disease (32). Research has shown that polyunsaturated fatty acids exhibit antibacterial activity by directly disrupting microbial cell membranes, stimulating the production of free radicals, and generating bioactive metabolites that lead to the elimination of microorganisms (33). In addition, supplementation with lactulose in high-protein diets was found to reduce the abundance of *Escherichia coli* and *Salmonella* in the cecum, an effect potentially mediated through the modulation of fatty acid metabolism.

Finally, we performed K-means clustering analysis on differential metabolites. This study revealed that a long-term high-protein diet reduced the levels of several metabolites, including carnitine, glutamic acid, arginine, tyrosine, and lysine, an adverse effect that was counteracted by lactulose supplementation, which increased their concentrations. Carnitine and amino acid metabolites are critically involved in maintaining intestinal health in animals. A critical function of carnitine is to act as an essential cofactor in the *β*-oxidation of fatty acids. It mediates the critical transfer of fatty acyl groups from the cytosol into the mitochondrial matrix, thereby enabling their oxidation to proceed and ultimately supporting the cell’s energetic demands (34). This process is of great significance for the energy supply and overall metabolic balance of intestinal cells. As fundamental building blocks of enterocytes, amino acids orchestrate cellular renewal cycles and tissue restoration pathways essential for intestinal architectural stability and absorptive capacity optimization (35). Arginine enhances antioxidant defense by elevating glutathione (GSH) levels, which helps scavenge reactive oxygen species (ROS), thereby strengthening intestinal antioxidant capacity and mitigating oxidative damage and related intestinal disorders (36). Within the intestine, lactulose undergoes microbial fermentation, leading to the production of short-chain fatty acids (SCFAs). These SCFAs exhibit significant antioxidant properties, which play a crucial role in mitigating oxidative stress in the intestinal environment (37). Tyrosine is a precursor of synthetic neurotransmitters (such as dopamine and norepinephrine) that affect intestinal motility and secretion through the gut-brain axis (38). Glutamate contributes to the maintenance of intestinal mucosal homeostasis and the promotion of epithelial cell proliferation through modulation of the mTOR signaling pathway (39). Lysine has been demonstrated to significantly stimulate B lymphocytes to proliferate and differentiate into plasma cells, which subsequently secrete specific antibodies (40). These antibodies recognize and bind with high affinity to antigenic determinants on pathogens, leading to neutralization, opsonization, or complement activation. As a result, pathogens are rendered inactive or marked for elimination by phagocytic cells, thereby augmenting the overall immune competence of the organism. These are the specific manifestations of the effect of lactulose on the intestinal health of geese, which improves intestinal health by promoting intestinal cell metabolism, improving intestinal antioxidant capacity and immune response.

## 5 Conclusion

In a 42-day feeding trial, dietary lactulose improved growth performance, intestinal health and nitrogen metabolism in geese. Specifically, lactulose increased the average daily gain of geese and reduced serum uric acid levels. In the ileum, lactulose increased villus height and the ratio of villus height to crypt depth. In the cecum, we observed a decrease in the content of *Escherichia coli* and *Salmonella* regulated by lactulose. Untargeted metabolomics converged with these phenotypes, showing pathway enrichment in glycerophospholipid and fatty-acid metabolism together with amino-acid metabolism recovery (notably Arg, Glu, Tyr, Lys). Metabolomic analysis revealed that lactulose effectively suppressed the proliferation of pathogenic intestinal bacteria through modulation of fatty acid metabolism. Concurrently, it contributed to the maintenance of intestinal barrier integrity by influencing glycerophospholipid metabolism, thereby mitigating the incidence of intestinal inflammatory responses. This study deepened our understanding of lactulose’s role in the metabolic processes of geese and provided novel strategies for modulating their intestinal health.

## Data Availability

The original contributions presented in the study are included in the article/supplementary material, further inquiries can be directed to the corresponding author.
